# Unmet needs and nursing home placements in Black, Latino, and White people living with dementia

**DOI:** 10.1002/alz.70265

**Published:** 2025-06-25

**Authors:** Jasmine Travers Altizer, Shivani Shenoy, Anisha Balaji, Marissa Bergh, Aasha Raval, Andreina Jimenez

**Affiliations:** ^1^ Rory Meyers College of Nursing, New York University New York New York USA; ^2^ School of Global Public Health, New York University New York New York USA

**Keywords:** barriers, community, inequity, long‐term care, older adults, unmet needs

## Abstract

**BACKGROUND:**

Many nursing home (NH) placements are avoidable considering equivalent care is possible in community settings.

**METHODS:**

This qualitative descriptive study explored the unmet needs and related barriers that drive avoidable NH placements among Black, Hispanic/Latino, and White persons living with dementia (PLWD). Interviews were conducted with PLWD, family care partners, and NH staff, from two New York NHs along with aging policy experts.

**FINDINGS:**

Seven distinct themes specific to unmet needs emerged from interviews including (1) assistance with activities of daily living and basic home maintenance; (2) resources and services; (3) treatment‐related services; (4) socialization; (5) individual preferences; (6) function of the home (e.g., unaddressed home modification needs); and (7) equipped, available, and supported family. Barriers contributing to unmet needs included awareness, knowledge, availability, affordability, and acceptability of resources, services, and supports.

**DISCUSSION:**

Strategies to address these unmet needs and improve community‐based services may help reduce these NH placements.

**Highlights:**

- Many people living with dementia can remain at home with necessary supports.
- There is limited paid and unpaid assistance to support people living with dementia.
- Navigating the complex landscape of services in the community is challenging.

## 1 BACKGROUND

The United States has seen a continued surge in nursing home (NH) use among Black and Hispanic/Latino older adults over the past decade at 12% and 24%, respectively.^1^, ^2^ Simultaneously, the number of White older adults using NHs has declined by 3%.^1^, ^2^ These disparate shifts are largely attributed to avoidable NH placements—care that could be provided in the community. This trend coincides with a broader shift in long‐term care (LTC) policy favoring home and community‐based services due to high costs, potential NH harms, and the COVID‐19 pandemic's exacerbation of NH risks.^3^ Harms of avoidable NH placements include increased risks of infection, isolation, poor quality care, misalignment with residents’ preferences, and exorbitant costs to the health‐care system.^2^ Black and Hispanic/Latino older persons living with dementia (PLWD) are at the greatest risk of experiencing the negative impact of avoidable NH placements due to documented disparities in LTC delivery, such as a lower likelihood of receiving preventive care (e.g., pneumococcal vaccinations) and higher chance of experiencing poor health outcomes (e.g., pressure ulcers) in these facilities.^4^, ^5^, ^6^, ^7^, ^8^ Moreover, Black and Hispanic/Latino older adults (age 65+) also have an overall dementia prevalence of 14.7% and 12.9%, respectively, and increasingly use NH services at disproportionate rates, despite recent initiatives to meet the LTC needs of older adults in the community setting.^9^ This inequity is unrelated to changes in population estimates.^7^, ^8^, ^10^, ^11^ Critically, hundreds of thousands of NH placements among all racial and ethnic groups are unnecessary and thus potentially avoidable, as equivalent care has been shown to be delivered in the community setting.^12^, ^13^

### 1.1 Reasons for shifts in NH use

Reasons for shifts in NH use among Black and Hispanic/Latino PLWD are multi‐factorial, but not well researched. Researchers have theorized that shifts in the racial and ethnic composition of NHs are partially due to White older adults choosing assisted living instead of NH placements, thereby increasing NH bed availability for Black and Hispanic/Latino older adults.^1^, ^9^, ^11^ Moreover, a large body of research points to the changing dynamics in the traditional use of informal caregivers among Black and Hispanic/Latino older adults: Black and Hispanic/Latino older adults have experienced dramatic changes in the home, possibly reducing their access to informal support that traditionally fulfilled their LTC needs.^14^, ^15^ A recent literature review shows that disparities in NH placements can additionally be explained by differences in enabling and need factors (Andersen's Behavioral Model of Health Services Use), including fewer financial resources and less access to community LTC services (enabling) and worse health (need).^16^, ^17^ While prior research has examined which factors drive older adults to use LTC, it has not assessed the unmet needs preventing older adults from receiving care in specific settings (i.e., community) and has lacked a focus on PLWD and Black and Hispanic/Latino older adults.^18^ Furthermore, definitions of “unmet needs” are vague and inconsistent, focusing mainly on unavailable and inconsistent assistance with function‐ and services‐specific needs. Black and Hispanic/Latino PLWD have unmet needs that can be outside of the scope of services and support provided to them. For example, environmental characteristics such as lack of healthy food and poor housing quality may predict unmet needs. Thus, decreased availability of family and not wanting to burden them, differential access to paid care, and structural/systemic racism (e.g., residential racial segregation and discrimination) may further explain increasing disparities in NH use.^7^, ^19^, ^20^

**RESEARCH IN CONTEXT**

1. **Systematic review**: Many nursing home (NH) placements are avoidable. This study explored unmet needs and barriers driving avoidable NH placements among Black, Hispanic/Latino, and White people living with dementia (PLWD).
2. **Interpretation**: Participants described multifaceted factors contributing to the unmet needs driving avoidable NH placements. Strategies to address these needs and improve understanding of the home and community‐based services landscape may help reduce these placements.
3. **Future directions**: Further research is needed to develop and evaluate interventions to address the identified unmet needs and barriers. Additionally, studies examining the implementation and scalability of these interventions in diverse communities would be valuable.

In this study, we operationalized “unmet need” as non‐receipt of any services, resources, or care that the PLWD needed to sustain their living within a community setting, but did not have as shared by the PLWD, family care partner, or a key informant. Identifying the unmet needs specific to the community setting driving avoidable NH placements among Black, Hispanic/Latino, and White PLWD is an actionable first step to reduce poor health outcomes and rising health‐care costs associated with avoidable NH placements. The aim of this study was to explore unmet needs and related barriers driving avoidable NH placements among Black, Hispanic/Latino, and White PLWD.

## 2 METHODS

### 2.1 Study design

This work is phase one of a larger, two‐phase mixed methods study that aims to design and psychometrically test an assessment instrument that measures unmet needs of PLWD in the community setting. The resulting data from the instrument will enable us to identify which unmet needs are most associated with avoidable NH placements among Black and Hispanic/Latino PLWD, compared to their White counterparts. In phase one, described herein, we used a qualitative one‐on‐one interview design to identify the unmet needs and barriers driving avoidable NH placements among Black, Hispanic/Latino, and White older adults to inform the development of the assessment instrument. One‐on‐one interviews are recommended for PLWD and hard‐to‐reach populations.^21^, ^22^ We chose to focus on NH placements that had already taken place while in a future study we will be able to focus on the trajectory of an avoidable NH placement using findings from this work.

Drawing on existing relationships, two large urban facilities in New York City were conveniently selected to be study sites. The two NHs belonged to a non‐profit corporation that served an urban population and provided community and residential care, such as NH care, short‐term rehabilitation, and hospice care.

### 2.2 Definitions

We defined avoidable NH placement and unmet needs to aid in the conceptualization of these issues.

#### 2.2.1 Avoidable NH placement

An avoidable NH placement was defined as a NH admission that could be prevented if equivalent care were provided in the community; that is, not in a NH for a skilled or chronic care need (e.g., ventilator care, tracheostomy, parenteral therapy, etc.) or for severe cognitive impairment.

#### 2.2.2 Unmet need

Unmet need was defined as non‐receipt of any services, resources, or care that was required by PLWD to sustain their living within a community setting.

### 2.3 Sample and eligibility criteria

Within these sites, we sought individuals who had experience and knowledge about the unmet needs that Black, Hispanic/Latino, and White PLWD experience that prevent them from remaining in their community. Therefore, our recruitment centered on a purposive sample of PLWD in an avoidable NH placement, family care partners (FCPs), and key informants (i.e., NH staff and aging policy experts).

#### 2.3.1 PLWD

Only PLWD ≥ 65 years of age admitted to the NH within the past 3 years with a Brief Interview for Mental Status (BIMS) score ≥ 8 (indicating no‐to‐moderate cognitive impairment) who could hear and speak clearly and who identified as Black, Hispanic/Latino, or White with an avoidable NH placement as defined above, were included. The BIMS score measures cognitive impairment level and ability to recall and provide valid information.^23^ Persons with mild‐to‐moderate cognitive impairment have been found to provide accurate and reliable responses about demographics and basic preferences.^24^ Dementia diagnoses, BIMS scores, and information on skilled or chronic care were ascertained via NH medical records by NH leaders and were provided to our research team who made the final determination on eligibility. PLWD did not need to perceive their NH placement as avoidable.

#### 2.3.2 FCPs

FCPs were family members who had an association with a PLWD at the NH site who met the aforementioned eligibility criteria. The participation of FCPs was not dependent on the PLWD's participation, and vice versa. The FCP did not have to be Black, Hispanic/Latino, or White.

#### 2.3.3 Key informants (NH staff and aging policy experts)

We targeted key informants who had direct knowledge of what residents’ home and caregiver situation was like prior to NH entry (e.g., care managers, social workers, occupational therapists, etc.). Thus, the only eligibility criteria for NH staff and aging policy experts were their ability to discuss the unmet needs experienced by Black, Hispanic/Latino, and White PLWD that prevented them from staying in the community.

All participants had to be able to hear; respond to questions in English or Spanish; and attend a phone, virtual, or in‐person interview.

### 2.4 Recruitment

#### 2.4.1 PLWD

NH leaders identified English‐ and Spanish‐speaking PLWD who met the eligibility criteria. Informed consent was completed prior to the interview with the FCP of the PLWD, and assent was completed with the PLWD. PLWD were screened using a six‐item screener to assess their ability to understand questions and follow study protocol.^25^

#### 2.4.2 FCPs

FCPs identified by the NH leaders were mailed an informational flyer by the NH site requesting their participation, along with an opt‐out consent for their PLWD to participate. A second round of mailings was implemented a few months later. NH leaders also shared FCPs' contact information with the research team. FCPs were contacted through phone and text messaging to facilitate recruitment.

#### 2.4.3 Key informants (NH staff and aging policy experts)

An informational session was held with NH executive leaders and NH staff to share study information and recruit eligible staff participants. During the session, a signup sheet was distributed to identify NH staff interested in participating in the study as a key informant. NH executive leaders also promoted staff participation by distributing flyers and sending a site‐wide e‐mail. Snowballing techniques were implemented to facilitate the recruitment of NH staff. Participants interviewed were asked to recommend eligible colleagues who might be interested in participating.

Aging policy experts were recruited from a national sample of (1) statewide aging organizations, including LeadingAge and area agencies on aging; (2) advocacy groups such as Center for Medicare Advocacy; and (3) health services researchers and leaders in aging and long‐term policy, with contact information identified via the organization's website. Twenty‐three aging policy experts were sent recruitment e‐mails by the research team and, like all potential participants, received information on the study aims, confidentiality procedures, incentives, and eligibility criteria virtually.

Aging policy experts, FCPs, and NH staff were pre‐screened to confirm their ability to speak about the unmet needs that drive avoidable NH placements for PLWD.^26^ All participants who met the eligibility criteria and verbally consented were invited for a one‐on‐one interview.

Prior literature suggests that data saturation occurs at ~15 one‐on‐one interviews^27^; however, because our groups were heterogeneous with potential for some interviews to be lacking in data because of the varying cognitive levels of the PLWD, we oversampled to ensure data saturation would be reached.^28^ Consented/assented PLWD participants each received a $20 NH credit and socks upon completion of the one‐on‐one interview sessions. Because PLWD participants reside in NHs and have limited use for monetary incentives, we provided the socks as a meaningful incentive alongside the $20 NH credit. Consented key informants and FCPs received a $50 Visa gift card upon completion of the interview sessions. FCPs’ participation incentive was increased from $30 to $50 to enhance recruitment efforts. NHs received a $1500 honorarium each for their participation. The institutional review board (IRB) of New York University approved the study.

### 2.5 Interview guide

The research team developed semi‐structured, open‐ended interview guides tailored individually for PLWD, FCP, and key informants (NH staff and aging policy experts; see supplementary file alz70265‐sup‐0001‐Appendix in supporting information). These guides were subsequently translated into Spanish for Spanish‐speaking PLWDs and FCPs. The translation process adhered to standards set forth by the IRB, were developed and validated by a third party, and certified by the IRB, ensuring the accuracy and reliability of the translated materials. With our interview guide, we aimed to solicit information about unmet needs that drive avodiable NH placements among Black, White, and Hispanic/Latino PLWD, therefore each interview guide was informed by Diwan & Moriarty's Conceptual Framework for Identifying Unmet Health Care Needs, literature review, and experts in the field.^29^ Questions increased in specificity and assessed unmet needs using domains from Diwan & Moriarty's framework and their accompanying barriers (e.g., can you tell us about anything you needed in the community but did not have? Please describe the biggest obstacles to accessing needed resources while living in the community). We aimed to ensure the questions were objective and without bias. The guide was shared with qualitative experts, PLWD, and aging policy experts for feedback and to test for clarity. Their feedback was incorporated into the final iteration of the interview guide.

#### 2.5.1 Diwan & Moriarty's Conceptual Framework for Identifying Unmet Health Care Needs

Diwan & Moriarty's conceptual framework aims to address unmet “health care” needs and provides direction for assessing barriers that may contribute to unmet needs beyond health care.^29^ While health is a multidimensional concept, Diwan and Moriarty acknowledge and address some ecological factors in the framework that may contribute to unmet needs. Thereby, unmet needs are categorized into six domains (basic maintenance, supportive, rehabilitative, treatment, preventive, and promotive) focused on “holistic” and overall well‐being. Basic maintenance are services such as food and shelter that are required to survive. Supportive services maintain functioning such as transport and homemaking, whereas rehabilitative services improve functioning and may include physical therapy, recreational services, and so forth. Medical services and clinical nursing care, that addreses illnesses are considered treatment while immunizations and other measures that reduce the risk of disease are defined as preventative. Last, promotive services improve well‐being, such as stress management.^29^

Additionally, factors that may hinder uptake of health services and/or affect the quality of life are defined as barriers to use. Within the domain of barriers, five factors may contribute to unmet needs (i.e., recognitions of need/knowledge about services/resources, service/resource availability, service/resource accessibility, service/resource affordability, and service/resource acceptability).^29^ In the context of the current study, the framework suggests that older adults experience unmet needs in these categories that prevent them from remaining in the community to receive LTC. Further, the framework enables the assessment of unmet needs across domains at different levels, allowing for a better understanding of the extent to which these needs are fulfilled.[Table alz70265-tbl-0001]

### 2.6 Demographic questionnaire

The demographic questionnaire included information specific to the participant such as age, sex, gender, race and ethnicity, country of origin/nationality, native language, length of time living in the United States, education, Medicaid status (PLWD), relationship to the PLWD (FCP), length of time residing in the NH (PLWD), or length of time employed at the NH or aging organization (NH staff and aging policy experts). All demographic questionnaires were completed by the participants themselves.

### 2.7 Procedure

One‐on‐one interviews with the PLWD took place in a private room at the NH. One‐on‐one interviews with aging policy experts, FCPs, and NH staff occurred via Zoom, telephone, or NH site, depending on the preference of the participant and participant type. All interviews were conducted between March 2022 and November 2022. Participants were asked to complete the brief 5 minute demographic questionnaire before the interview. One of four researchers from the research team conducted each interview. All were trained in qualitative research methods. Each interviewer was shadowed by another researcher for their first interview to provide feedback and thereby standardize the interview technique and ensure fidelity. Interviews lasted ~30 minutes and were audio‐recorded, professionally transcribed verbatim, and reviewed for accuracy by the research team. To ensure the adequacy and quality of the data, the team applied interview techniques that maximize completeness and truthfulness.^30^

### 2.8 Data analyses

Qualitative data were analyzed iteratively using hybrid inductive and deductive thematic analysis. This hybrid approach enabled the integration of theory‐driven coding while allowing for data‐driven construction of themes.^31^, ^32^ Initial coding categories were developed deductively based on Diwan & Moriarty's framework, relevant literature, and a preliminary transcript review. The research team independently coded a subset of transcripts, refining code definitions collaboratively. The principal investigator oversaw codebook development, ensuring consistency across coding. NVivo 12 qualitative data analysis software was used to facilitate data coding and tracking to ensure data dependability.^33^ The research team reviewed the codes, identified patterns among them, and inductively constructed themes. Themes and discrepancies were reconciled during weekly team meetings. This thematic analysis concluded when no new themes emerged. Last, the identified themes were “defined and refined” to capture the “essence” of the data it represents.^34^ The Consolidated Criteria for Reporting Qualitative Research (COREQ) reporting guidelines were followed to report this study (see supplementary file alz70265‐sup‐0003‐COREQ in supporting information).

## 3 FINDINGS

In total, 61 interviews were conducted: 19 PLWD, 17 FCP, and 25 key informants (15 NH staff and 10 aging policy experts). The majority of FCPs (71%) and key informants (80%) were female. PLWD participants were more evenly distributed on sex (male = 53%, *n* = 10; female = 42%, *n* = 8; and prefer not to say = 5%, *n* = 1). Participants mostly identified as non‐Hispanic White and Hispanic/Latino across participant type. Total 11% (*n* = 2), 12% (*n* = 2), and 8% (*n* = 2) of PLWD, FCP, and key informants identified as Black, respectively, whereas 32% (*n* = 6), 47% (*n* = 8), and 32% (*n* = 8) of PLWD, FCP, and key informants identified as Hispanic/Latino, respectively. Out of the 17 FCP participants, 12 were the children of a PLWD, four were their nieces and nephews, and one was a spouse. The majority of PLWD completed no more than a high school degree (78%, *n* = 14), whereas the majority of FCP (82%, n = 14) and key informants (92%, n = 23) had more than a high school diploma (Table 1).

**TABLE 1 alz70265-tbl-0001:** Participant demographics and characteristics.

	PLWD *n* (%)	FCP *n* (%)	Key informants (NH staff & aging policy experts) *n* (%)
**Age in years** (Mean, SD)	82.4, 8.9	58.6, 11.3	–
**Sex**			
Female	8 (42.1)	12 (70.6)	20 (80)
Male	10 (52.6)	5 (29.4)	5 (20)
Prefer not to answer	1 (5.3)	0	0
**Race and ethnicity**			
Non‐Hispanic/Latino Black	2 (10.5)	2 (11.8)	2 (8)
Non‐Hispanic/Latino White	10 (52.6)	7 (41.2)	14 (56)
Hispanic/Latino, Native American, or American Indian	0	0	1 (4)
Hispanic/Latino (White and other)	6 (31.6)	8 (47)	8 (32)
Hispanic/Latino Black	1 (5.3)	0	0
**Education**			
Less than high school diploma	6 (33.3)	0	0
High school diploma or GED	8 (44.4)	3 (17.6)	2 (8)
Some college	1 (5.6)	6 (35.3)	2 (8)
Associate's degree	1 (5.6)	1 (5.9)	2 (8)
Bachelor's degree	2 (11.1)	5(29.4)	4 (16)
Master's degree	0	1 (5.9)	8 (32)
Professional degree	0	0	7 (28)
Doctorate	0	1 (5.9)	0
**Lived in the US**			
1–5 years	2 (10.53)	0	0
Over 20 years	17 (89.5)	17 (100)	25 (100)
**Relationship with PLWD**			
Child	–	12 (70.6)	–
Niece/nephew	–	4 (23.5)	–
Spouse	–	1 (5.9)	–
**Medicaid enrollment status**			
No	6 (31.6)	–	–
Yes	8 (42.1)	–	–
Unsure	5 (26.3)	–	–
**Length of stay in NH**			
1–5 years	12 (63.2)	–	–
< 1 year	7 (36.8)	–	–
**Length of employment at current facility/organization**			
1–5 years	–	–	4 (16)
11–15 years	–	–	4 (16)
16–20 years	–	–	2 (8)
6–10 years	–	–	3 (12)
< 1 year	–	–	4 (16)
> 20 years	–	–	8 (32)
**Country of origin/nationality**			
North America/Central America	9 (47.4)	–	–
Europe	2 (10.5)	–	–
Caribbean islands	4 (21)	–	–
Other	3 (15.8)	–	–
Prefer not to say	1 (5.3)	–	–
**Native language**			
English	12 (63.2)	13 (76.5)	21 (84)
Spanish	7 (36.8)	4 (23.5)	4 (16)
	**19 (100)**	**17 (100)**	**25 (100)**

Abbreviations: —, data not collected; FCP, family care partner; GED, Graduate Equivalency Degree; NH, nursing home; PLWD, person living with dementia; SD, standard deviation.

### 3.1 Themes from analysis

The five domains from Diwan & Moriarty's framework (basic maintenance, supportive, treatment, rehabilitative, and promotive) and the five barriers (recognition of need/knowledge, availability, accessibility, affordability, and acceptability of resources) remained emergent in qualitative analysis. Thematic analysis of the domains gave rise to seven themes from the data: (1) assistance with activities of daily living (ADLs) and basic home maintenance; (2) resources and services; (3) treatment‐related services; (4) socialization; (5) individual preferences; (6) function of the home; and (7) equipped, available, and supported family. These themes centered around the barriers defined within Diwan & Moriarty's framework that contribute to unmet needs: awareness, knowledge, availability, affordability, and acceptability of resources, services, and supports. Descriptions of each theme with exemplar quotes can be found in Table 2.

**TABLE 2:** Themes and exemplar quotes of unmet needs.

Theme	Quotes
**Assistance with Activities of Daily Living and Basic Home Maintenence**	“They need somebody to accompany them everywhere. To the doctors, to the dentist, they need somebody to make their schedules. They need somebody to prepare their meals. Basically you need somebody all the time.” (FCP #1, Black, non‐Hispanic/non‐Latino)
	“So I mean, I think that's the biggest factor is the support system, informal and also formal. So a lot of people fall in between. They're not rich enough to get private help and they're not poor enough to get Medicaid.” (KI #18, White, non‐Hispanic/non‐Latino)
**Resources and Services**	“I think that the other thing we see is that in some communities that are generally lower income, there might not be the available resources in that community for people to access.” (KI #22, White, non‐Hispanic/non‐Latino)
	“They have no idea. Their families, they have no idea. Like I'll talk to somebody, and then I say, well, what about PACE? Maybe you could keep them at home, PACE during the day, they could be here, and it's supplemented to at‐home care in the evening hours and stuff, if you even want that, but at least they're safe during the day. They say “I didn't know about that,” and I go… That's why the outreach has to be out there consistently.” (KI #6, White, Non‐Hispanic/Non‐Latino)
**Treatment‐Related Services**	“The family doctor have to be checking on this person per se, all the time. In whatever changes the family members see, they have to always write it down. And then when the doctor comes, I'll say, ‘Oh, doctor, my mother was fine last week, but I see a little bit changes this week. What's going on?’” (KI #5, Black, non‐Hispanic/non‐Latino)
	“I need doctor's appointments.” (PLWD #19, Black, non‐Hispanic/non‐Latino)
	“Transportation is a problem for people if they need to get to the doctor. How do you get groceries? How do you get medicine? I mean, how do you get the things you need if you're living at home? So even if you have good home care, they're not going to bring the medicine to you. How do you get it?” (KI #13, White, non‐Hispanic/non‐Latino)
**Socialization**	“It goes back to having someone coming into the home and spending time with them and socializing with them. Some of them don't get to socialize at all.” (KI #15, other/unknown, Hispanic/Latino)
	“There was a downfall to everything, but I do think that socialization and support from the community, whether it's a community center, a religious organization. I think it would help because many elderly people are alone.” (FCP #12, White, non‐Hispanic/non‐Latino)
**Individual Preferences**	“No, I want my own time, my own space, my own house…. And when it comes to a time that I can't do it, I don't want to live with the kids or go to a nursing home.” (PLWD #5, White, non‐Hispanic/non‐Latino)
	“The thing is that his apartment where he sleeps and all of that, is full… He has proposed it, but I don't want to be a burden.” (PLWD #16, Black, Hispanic/Latino)
**Function of the Home**	“Sometimes when you have dementia, you could walk around. But it also goes after the physical part of you.” “There're steps in the home and then maybe they can't afford to get a StairMaster to go up and down the steps. There's no bath down on entry level. So, the physical makeup of the home is one part, the economics, and the help.” (KI #6, White, non‐Hispanic/non‐Latino)
	“They may need treatments or wound care that a caregiver then or someone would have to provide for them. They may need medical equipment in their homes, such as a special bed or walker or wheelchair or cane.” (KI #11, White, non‐Hispanic/non‐Latino)
	“She had stairs to go into the apartment. It was a one‐bedroom apartment, small. It's not wheelchair accessible, it's not handicapped accessible. There was I think 13, 14 steps to get up into the apartment.” (FCP #10, White, non‐Hispanic/non‐Latino)
	“I had help most of the time, but I did most of it. But I had just problems with the steps, because they were right down, like five or six steps.” (PLWD #13, White, non‐Hispanic/non‐Latino)
**Equipped, Available, and Supported Family**	“I would say people to come in and provide care because I feel that when you are the child of… Especially that was my father and I'm the daughter that they don't like assistance with bathing and dressing because they feel shame.” (FCP #1, Black, non‐Hispanic/non‐Latino)
	“I think another thing that's needed is training … family caregivers, too, to help them understand the symptoms of dementia and the symptoms of behavioral problems, that they know why they're happening and how to prevent them and how to manage them.” (KI #11, White, non‐Hispanic/non‐Latino)

### 3.2 Assistance with ADLs and basic home maintenance

Participants commonly discussed challenges with securing assistance for caregivers and PLWD such as preparing meals, getting groceries, and acquiring supportive services such as home health aides, homemakers, and social workers. These challenges were rooted in limited awareness, knowledge, availability, affordability, and acceptability of the assistance.

Related to limited availability of supports, participants expressed the importance of having a paid or unpaid caregiver to support PLWD living in the community for more time than is currently provided. As one key informant shared,

“*If you are a person who is working and you have your loved one at home with a 4‐h home attendant, when the home attendant leaves, your loved one is by themselves until you get home from work, and that's a big issue.” (KI #15, other/unknown, Hispanic/Latino)*

Participants further discussed PLWD requiring 24 hour care or supervision, which is often burdensome for FCPs who need to work or run errands and leave their family member unattended. In some cases, assistance (e.g., home health aide) was not available due to staffing issues. FCPs specifically mentioned that the lack of available home health aides was a driver of NH placement, as they could not access the skilled care their family member needed.

Affordability was another significant challenge that affected the type and amount of assistance to which a PLWD and/or FCP had access. For example, participants mentioned that home health services could only be provided as a part of Medicaid, and only a specific number of hours were allocated to the PLWD per day. As a result, PLWD that did not qualify for Medicaid or did not have sufficient hours allocated experienced challenges in remaining in the home setting. An FCP described it as such:

*“They wouldn't even offer my dad an aide, because I asked his doctor and his doctor said that he didn't qualify. So, I didn't understand what that was about, because what do you have to be to qualify?” (FCP #3, White, Hispanic/Latino)*

Even when assistance was available and affordable, some FCPs and PLWD were not accepting of the assistance; they mentioned feeling uncomfortable with home attendants visiting their homes.

*“Part of the issue is the older adult being willing to accept it (support). She (PLWD) would fire the caregivers because she didn't like them or didn't like the way they were acting or didn't think that they were doing anything.” (KI #22, White, non‐Hispanic/non‐Latino)*

It was apparent that family members were critical to the assistance provided to the PLWD. One FCP shared, for example,

*“I prepared his food. I was taking care of him. So another person in the house, I couldn't see what they would be doing.” (FCP #2, White, non‐Hispanic/non‐Latino)*

### 3.3 Resources and services

Participants discussed variability in the accessibility and availability of community resources and services—such as senior centers, day cares, and meal programs—along with the quality of care provided within these resources and services and the acceptance of them. For example, when asked why a PLWD did not eat the food provided, the PLWD replied, “because the food is not Hispanic,” demonstrating lack of acceptability (PLWD #8, White, Hispanic/Latino).

Additionally, participants described the inability to access supportive services such as meal programs and transportation due to the complexity of insurance systems. They were unaware of what services were covered by insurance, which led to reluctance to seek or accept sufficient support.

Participants further highlighted that the neighborhood or community in which they live can play a crucial role in availability of resources, as lower income communities are likely to have fewer resources and services available (Table 2).

When community centers were available, participants noted the additional supports that they provided such as breakfast and lunch but reported that the centers were only open for limited hours (e.g., 8 am–noon).

### 3.4 Treatment‐related services

For PLWD with comorbidities, treatment‐related services such as routine doctors’ appointments, medications, and acute care services were especially important. Participants highlighted lack of transportation, inability to use public services, scheduling difficulties, and lack of skilled staff as insufficient treatment‐related services, which resulted in PLWD missing important doctor's appointments, routine medications, and treatment. PLWD who had diabetes or other comorbidities required routine glucose monitoring and insulin shots, which was not possible if a skilled staff was unavailable, for example,

*“If you're on insulin, and there's nobody there, the way it is, they don't send a nurse in to do that. That's a skilled need. So, there's nobody there to give the insulin.” (KI #6, White, non‐Hispanic/non‐Latino)*

Key informants and FCPs additionally discussed difficulties in administering medications for PLWD when PLWD are tasked to do so on their own. Errors become increasingly prevalent, as PLWD often find it difficult to open medication bottles, forget to take medications when stored in medication dispensers or organizers, and may take the wrong quantity of medication. Thus, FCPs must regularly remind the PLWD to take medications and observe them to ensure it is taken correctly. This is difficult, given that PLWD might exhibit behaviors related to delirium and aggression. A key informant described this tension, stating that,

*“They don't take their medications. Their loved ones are trying to force them. They don't know what to do to get them to take the medication.” (KI #15, other/unknown, Hispanic/Latino)*

### 3.5 Socialization

A few participants mentioned concerns about lack of socialization for PLWD, articulating that PLWD often feel lonely and could benefit from social support from adult day centers, religious organizations, and senior centers. In instances where the PLWD lives alone, participants mentioned the need for someone to spend time with PLWD.

*“Their mind, their memory has dysfunction, because of their age, and they need people to talk to. If they're alone in the home, of course, depression is going to become worse.” (FCP #9, White, non‐Hispanic/non‐Latino)*

In contrast, some FCPs mentioned that their family members used to be active and visit friends, but socialization declined with cognition, and they no longer interact with anyone. When asked if their family member participated in activities organized by senior centers, an FCP shared,

*“…he didn't participate a lot there. He would never play pool or dominos. Sometimes I would come to pick him up and he'd be sitting outside and in his own world.” (FCP #6, other/unknown, Hispanic/Latino)*

### 3.6 Individual preferences

Some individuals preferred to not access some or all long‐term services and supports. For example, some FCPs and PLWDs simply preferred to not receive care from home health aides. This may have been because they preferred no intrusions into their space, were averse to assistance from others, or did not trust home health aides. Furthermore, some PLWDs shared that there was simply no space for a home health aide in their house. One PLWD mentioned that they liked to do daily activities, such as cooking, themselves:

*“I liked to cook. I would make oatmeal, and sometimes pancakes for breakfast; sandwiches for lunch.” (PLWD #18, other/unknown, Hispanic/Latino)*

### 3.7 Function of the home

Function of the home refers to how well one's home was accommodating to the needs of the PLWD, overall safety concerns in the home, and the need for home modifications.

Key informants and FCPs discussed safety conditions extensively. For instance, many participants shared that PLWDs’ home environment had stairs and lacked handrails and shower bars, which posed a great risk for falls. The inability to avoid a fall or support the PLWD after a fall was a major concern, often leading to NH placements per the doctor's recommendation. An FCP shared,

*“I would say the last year he fell every other week. He was falling and they would take him to the hospital.” (FCP #2, White, non‐Hispanic/non‐Latino)*

For PLWD, the inability to modify home environments was a significant barrier to remaining in the community. Many reported that they had issues with “the steps” and that their home did not accommodate a wheelchair. Additionally, participants shared that the safety conditions within their homes could be harmful to PLWD when they are living alone.

“*They might start off doing something and then they forget. They leave the water running. The stove is on. So, conditions are very harmful to someone who has dementia to be living at home alone.” (KI #15, other/unknown, Hispanic/Latino)*

### 3.8 Equipped, available, and supported family

The equipped, available, and supported family theme pertained to the nuances of the familial relationships between FCP and PLWD; how involved family members were in taking care of the PLWD; and the PLWD's emotional needs and concerns that the family sought to address. Participants particularly mentioned the interpersonal difficulties that appeared when FCPs assisted the PLWD with ADLs. They mentioned that difficulties arise due to parent–child or spousal relationships, which could lead to the PLWD feeling shame or opting out of care. Likewise, some PLWD shared that they preferred to stay at home but could not because of troubled relationships with family. A PLWD mentioned,

*“My daughter and I sometimes, we just didn't have a calm, harmonious relationship. That was the only thing. In fact, that's one of the reasons why I'm in this nursing home instead.” (PLWD #2, Black, non‐Hispanic/non‐Latino)*

Some key informants shared that there is a need to train and provide knowledge on resources available to reduce burden for FCPs, especially given that FCPs have consistently reported that a lack of knowledge and education about dementia care is a consistent concern. FCPs in this study reported that they often do not know where to obtain knowledge about caring for PLWD and where to find support when their family member is diagnosed with dementia. An FCP mentioned,

*“There is not one specific source that you can go to find out all of these things. There's not one central resource that you can actually go to say, ‘Hey, I need….’ You have to find the resources yourself, nobody comes and gives you a handbook and says, ‘Hey, your parent has dementia, do this, do that.’” (FCP #1, Black, non‐Hispanic/non‐Latino)*

## 4 DISCUSSION

In this study, themes around unmet needs that drive avoidable NH placements among Black, Hispanic/Latino, and White PLWD centered around the limited availability, knowledge, awareness, access, and acceptability of resources, services, and supports for these groups, along with the PLWD's preferences for support. Participants described the multifaceted barriers Black, Hispanic/Latino, and White PLWD face while living in their communities, that ultimately contribute to avoidable NH placements. These barriers ranged from lack of paid and unpaid assistance to trouble navigating the complex landscape of services in the community. To our knowledge, this is the first study that aimed to identify the unmet needs that drive NH placements in the United States among a racially and ethnically diverse group of PLWD.

Our findings reflect the piecemeal nature of assistance that is available for PLWD, who often rely on complex and precarious caregiving networks composed of family, friends, and paid assistance.^35^ For example, all three groups (PLWD, FCPs, and key informants [i.e., NH staff and aging policy experts]) emphasized that the lack of available assistance with home maintenance activities such as transportation, meal preparation, finances, grocery shopping, cleaning, and laundry, and ADLs such as eating, dressing, and toileting was a major contributor to a NH placement. Before NH admission, this assistance was commonly provided by unpaid care partners, paid caregivers, homemakers, and home health aides, who were not always readily available. In recent years, it has been shown that adult children, nieces, and nephews are more likely to be employed and/or have children of their own, reducing their availability to provide PLWD with the care necessary to keep them safe and healthy.^36^ Furthermore, paid caregivers were described as unreliable, having limited or no available hours, and having had little proper training. Participants reported that this caused strain on the FCPs’ ability to work conventional full‐time schedules. In addition to assistance with ADLs and instrumental ADLs, some PLWD required continuous supervision for safety purposes to prevent wandering or injury; this is the case for many PLWD experiencing cognitive impairment. This can necessitate nearly around‐the‐clock care or observation, which is increasingly challenging for FCPs to provide, especially when faced with multiple responsibilities. FCPs discussing this burden expressed anxiety about the gaps in care for their family member and the challenges of managing caregiving when 24 hour care is needed.

Formal home and community‐based services (HCBS) offer vital alternatives to unpaid care when FCPs are unavailable and when the needs of the PLWD surpass what the FCP can provide. HCBS include transportation assistance, adult day services, meal delivery services, homemakers, personal care aides, and home health aides. Use of these services has been associated with delayed NH placement.^37^, ^38^ However, participants in this study noted that the transition of Black, Hispanic/Latino, and White PLWD to NHs often occurred when the PLWD's needs outpaced the services available or when services were unsuitable or unavailable, either due to lack of affordability, awareness, or acceptance of services, or by the limited hours approved by insurance. Therefore, there is a need to increase the availability, knowledge, awareness, access, and acceptance of these services.

Regarding lack of affordability, HCBS are covered primarily through Medicaid programs and thus are primarily available only to Medicaid beneficiaries. Without long‐term care insurance, many adults in the United States do not qualify for any financial assistance with HCBS. Multiple key informants reported that many older adults “fall through the cracks” because they are “not rich enough to get private help and not poor enough to get Medicaid” and cited this as a major problem with the long‐term care system (KI #18). Even when an individual is eligible for Medicaid, there are several barriers to obtaining sufficient formal support. For instance, these same key informants noted that even when services were available, bureaucratic barriers inherent within Medicaid (e.g., lack of information and/or long wait lists) hindered access and efficacy of these services. Further, many PLWD who are eligible to receive HCBS through Medicaid do not qualify for enough assistance to meet their care needs, potentially leaving them without appropriate care.

Another catalyst for avoidable NH placements among Black, Hispanic/Latino, and White PLWD described in this study was a major fall or repeated falls. Often, after a PLWD was sent to the hospital post fall and subsequently admitted to a NH for short‐term rehabilitation, their stay was frequently converted to long term because the PLWD was unable to return home or a health‐care provider recommended NH placement. FCPs often felt they had no choice but to send their family member to a NH for increased supervision. This finding is consistent with other studies reporting that falls, particularly those that result in injury, are significantly associated with NH placements.^39^ While falls are a major safety concern among PLWD, there are other interventions to promote safety in the home, such as increasing access to home modifications and durable medical equipment for which organizations such as area agencies on aging provide support.^40^ Moreover, providers should proactively identify early stages of frailty and incorporate proactive interventions, like exercise‐based programs and physical therapy, that promote strength and coordination to reduce fall risk.^41^

Another important component of avoidable NH placements commonly overlooked was the effect of loneliness on well‐being. Older adults reported increased loneliness as a result of COVID‐19, with families becoming more geographically distant.^42^ Indeed, the US Surgeon General marked loneliness as an even greater indicator of premature death than smoking 15 cigarettes every day.^43^

While this study did not explicitly examine how race and socioeconomic status may affect the unmet needs leading to avoidable NH placements for Black and Hispanic/Latino PLWD specifically, which will be done in the next iteration of this work, it is important to acknowledge the potential effects of structural racism on access to community resources that, in turn, may perpetuate avoidable NH placements. Studies have suggested that Black and Hispanic/Latino older adults have lower use rates of community‐based resources within Medicaid.^38^, ^44^ For example, in one study, Black HCBS recipients were less likely to receive home modifications, case management, and skilled nursing care than White recipients.^44^ Lower home modification rates are likely due, at least in part, to lower home ownership rates among Black older adults, the result of decades of housing policy discrimination.^45^ Further, provider bias may play a role in the type and quantity of services that are recommended to PLWD.^46^ Future research should focus on both structural and personal bias in receipt of community‐based resources for Black and Hispanic/Latino older adults.

## 5 CONCLUSION

Our findings can inform efforts to address avoidable NH placements among a highly vulnerable population.^7^ Addressing unmet needs will help ensure that all older adults have equitable access to preferred, person‐centered care. NH placements for PLWD are often considered inevitable. However, with access to appropriate and sufficient services, costly NH placements could be deferred or avoided altogether. It is important that these services are equitably distributed and culturally tailored for all older adults. It is also critical that providers work proactively to support PLWD, as early use of services is associated with prolonged ability to remain in the community. Preventing avoidable NH placements and enabling aging in the home is considered a priority by federal, state, and local entities. This is important in the context of a shortage of human resources for NH care and poor care delivery in NHs, especially for older adults of racial and ethnic minoritized backgrounds.^10^ Policy makers, aging agencies, researchers, and government entities need to understand how to keep older adults in the community for their long‐term care to maximize choice, control, and optimal health outcomes.

### 5.1 Limitations

There are a few limitations to note. Despite sampling PLWD who were recently admitted to NHs (< 3 years) and New York City's racial and socioeconomic diversity, recall during interviews may be unreliable, and findings may not be broadly generalizable. However, our sample size exceeded recommendations and we sampled until data saturation was reached to account for limited information gathered in previous interviews. We also included a national sample of aging policy experts and used existing literature and items from other scales to substantiate our findings.^47^ Another potential limitation is that we only interviewed PLWD in the NH, but in the future stages of the grant, we will be interviewing PLWD living in the community. Finally, the sample across racial and ethnic groups is too small to draw conclusions concerning racial and ethnic differences in unmet needs, but this will be possible in future stages of the grant through the assessment survey that was developed from these data.

## CONFLICT OF INTEREST STATEMENT

The authors have no conflicts to disclose. Author disclosures are available in the supporting information (see file alz70265‐sup‐0002‐ICMJE).

## CONSENT STATEMENT

All human subjects provided informed consent.

## Supporting information



Supporting Information

Supporting Information

Supporting Information
