# Supplementary material for: Unmet needs and nursing home placements in Black, Latino, and White people living with dementia
Source: Alzheimers Dement. 2025 Jun 25;21(6):e70265. doi: 10.1002/alz.70265 (PMC12198474; doi:10.1002/alz.70265)
Supplement: Supplementary file 1 — Supporting Information [file ALZ-21-e70265-s003.docx]

**Appendix: Interview Guide Questions**

| **Category** | **Interview Guide Questions** |
| --- | --- |
| **PLWD Specific** | 1. Please introduce yourself, tell me your story, and how you got to be in the nursing home. 2. Please describe the conditions in the home that may have led to you needing nursing home care. 3. Please tell me about your living environment prior to the nursing home. 4. Please describe your level of support in the community setting.  - Probe: What support would you have wanted/considered helpful?  1. Can you tell us about anything you needed in the community setting that you didn’t have? 2. Please tell me how you got your everyday needs met, such as food, while living in the community setting. 3. Please tell me how you accessed medications and treatments while living in the community setting. 4. Please tell me how you travel to see your providers/doctors while living in the community setting. 5. Please describe your knowledge of resources (e.g., senior living center, meals on wheels, transportation) that would have been helpful while living in the community setting. 6. Please describe the biggest obstacles to accessing needed resources (e.g., financial resources) while living in the community. 7. Please tell me about how you spent time doing things that you enjoyed and/or with people who you enjoyed. |
| **Key Informant (NH Staff/Aging Policy Expert) & FCP Specific** | 1. Please introduce yourself and tell me about your current role. 2. Please tell us what PWD typically need in the community, that they don’t have access to, which leads to avoidable nursing home placements.  - FCP Probe: Can you tell us how your family member (or the person you know) ended up in the nursing home? - Key Informant Probe: Can you tell us what was missing from the community that would have allowed them stay?  1. Please describe the conditions in the home that are typically unmet and lead to avoidable nursing home placements among PWD while living in the community setting? 2. Please describe the support systems that are typically lacking while living in the community setting among PWD?  - Probe: Can you tell us about the hours and reliability required for a paid caregivers  1. Please tell me how a PWD secure everyday needs, such as food, while living in the community setting?  - Probe: Can you tell us about how PWD get access to good nutrition or specific dietary restrictions such as low sodium, soft consistency or diet related to another disease such as diabetes.  1. Please tell me how a PWD accesses medications and treatments while living in the community setting? 2. Please tell me about unmet needs related to transportation to travel to health facilities among PWD living in the community setting? 3. Please describe the typical knowledge of available resources in the community among PWD? 4. Please describe the biggest obstacles to accessing needed resources while living in the community among PWD? |
| **FCP Specific** | 1. Please tell us about your other obligations or the accommodations that you need to provide care to your family member in the community. 2. Please tell us about your living environment. |
