# Supplementary material for: Unmet needs and nursing home placements in Black, Latino, and White people living with dementia
Source: Alzheimers Dement. 2025 Jun 25;21(6):e70265. doi: 10.1002/alz.70265 (PMC12198474; doi:10.1002/alz.70265)
Supplement: Supplementary file 3 — Supporting Information [file ALZ-21-e70265-s002.docx]

**COREQ (COnsolidated criteria for REporting Qualitative research) Checklist**

A checklist of items that should be included in reports of qualitative research

| **Topic** | **Item No.** | **Guide Questions/ Description** | **Author Responses** |
| --- | --- | --- | --- |
| **Domain 1: Research team and reflexivity** | | | |
| *Personal Characteristics* | | | |
| Interviewer/facilitator | 1 | Which author/s conducted the interview or focus group? | Jasmine Travers, Andreina Jimenez, Shivani Shenoy, Anisha Balaji,  Marissa Bergh, Aasha Raval |
| Credentials | 2 | What were the researcher’s credentials? E.g. PhD, MD | Jasmine Travers, PhD  Andreina Jimenez, MPH  Shivani Shenoy, MBA, MPH  Anisha Balaji, MPH  Marissa Bergh, BSN, RN  Aasha Raval, MPH  Sophie Levine, BA |
| Occupation | 3 | What was their occupation at the time of the study? | Jasmine Travers- Assistant Professor  Andreina Jimenez - Project Manager  Shivani Shenoy - Research Assistant  Anisha Balaji - Research Assistant  Marissa Bergh - Research Assistant  Aasha Raval - Research Associate  Sophie Levine – Research Assistant |
| Gender | 4 | Was the researcher male or female? | All Female |
| Experience and training | 5 | What experience or training did the researcher have? | The researchers had CITI training for Social and Behavioral Research and qualitative research training. |
| *Relationship with participants* | | | |
| Relationship established | 6 | Was a relationship established prior to study commencement? | No |
| Participant knowledge of the interviewer | 7 | What did the participants know about the researcher? e.g., personal goals, reasons for doing the research | The participants knew the researcher's occupation, affiliated organization, and research goal. |
| Interviewer characteristics | 8 | What characteristics were reported about the inter-viewer/facilitator? e.g., Bias, assumptions, reasons, and interests in the research topic | The researchers had an interest in the research. Jasmine Travers had previous experience working in nursing home research. |
| **Domain 2: Study design** | | | |
| *Theoretical framework* | | | |
| Methodological orientation and Theory | 9 | What methodological orientation was stated to underpin the study? e.g., grounded theory, discourse analysis, ethnography, phenomenology, content analysis | For the methodological orientation, the study used a directed content and thematic analysis approach to analyze the collected data. |
| Sampling | 10 | How were participants selected? e.g., purposive, convenience, consecutive, snowball | A purposive sample of two nursing homes had been recruited. The researchers contacted the leaders at nursing home who can assist in recruiting PLWD residents, family care partners, nursing home staff, and aging and policy-relevant stakeholders). |
| )Method of approach | 11 | How were participants approached? e.g., face-to-face, telephone, mail, email | PLWD residents were approached face to face in a private room at NH facility. FCPs, and NH staff interviews took place on zoom,  phone, or NH site depending on the preference of the participant and participant type. |
| Sample size | 12 | How many participants were in the study? | 61 |
| Non-participation | 13 | How many people refused to participate or dropped out? Reasons? | Three Family Care Partners (FCPs) declined to participate, while an additional 13 FCPs did not respond to our calls. Furthermore, three members of the nursing home (NH) staff did not meet the key informant threshold required for participation. Five Persons Living With Dementia (PLWD) declined to participate, and seven FCPs refused to provide consent on behalf of their respective PLWD. One PLWD had to leave the nursing home for skilled care, and 10 PLWDs did not pass the screening criteria. One PLWD passed away during the recruitment phase, and another PLWD left the nursing home during this process. Additionally, three FCPs were unavailable to provide consent for the PLWD they represented. One PLWD was not interviewed due to the transition to Aim 2. |
| *Setting* | | | |
| Setting of data collection | 14 | Where was the data collected? e.g., home, clinic, workplace | Part of the data was collected in Nursing Home for in-person interviews. For virtual interviews, data was collected from workplace. |
| Presence of non-participants | 15 | Was anyone else present besides the participants and researchers? | No |
| Description of sample | 16 | What are the important characteristics of the sample? e.g., demographic data, date | Sample characteristics are presented in the table 1. |
| *Data collection* | | | |
| Interview guide | 17 | Were questions, prompts, guides provided by the authors? Was it pilot tested? | Yes |
| Repeat interviews | 18 | Were repeat interviews carried out? If yes, how many? | None |
| Audio/visual recording | 19 | Did the research use audio or visual recording to collect the data? | Yes |
| Field notes | 20 | Were field notes made during and/or after the interview or focus group? | Yes |
| Duration | 21 | What was the duration of the interviews or focus group? | Duration of the interviews was maximum 30 minutes. |
| Data saturation | 22 | Was data saturation discussed? | Yes, data saturation was discussed in the method section. |
| Transcripts returned | 23 | Were transcripts returned to participants for comment and/or corrections? | No, transcripts were not returned to the participants. |
| **Domain 3: analysis and findings** | | | |
| *Data analysis* | | | |
| Number of data coders | 24 | How many data coders coded the data? | 4 |
| Description of the coding tree | 25 | Did authors provide a description of the coding tree? | No |
| Derivation of themes | 26 | Were themes identified in advance or derived from the data? | Themes were derived from the data. |
| Software | 27 | What software, if applicable, was used to manage the data? | NVivo |
| Participant checking | 28 | Did participants provide feedback on the findings? | No |
| *Reporting* | | | |
| Quotations presented | 29 | Were participant quotations presented to illustrate the themes/findings?  Was each quotation identified? e.g. participant number | Yes, quotations have been presented throughout result section, with participant codes assigned to all participants and used against quotations. |
| Data and findings consistent | 30 | Was there consistency between the data presented and the findings? | We endeavored to report the study findings in a clear, consistent manner to accurately reflect the data that have been collected. |
| Clarity of major themes | 31 | Were major themes clearly presented in the findings? | Yes, major themes are clearly presented in the result section. |
| Clarity of minor themes | 32 | Is there a description of diverse cases or discussion of minor themes? | Yes, all data relating to the themes are presented in results section. |

Developed from: Tong A, Sainsbury P, Craig J. Consolidated criteria for reporting qualitative research (COREQ): a 32-item checklist for interviews and focus groups. *International Journal for Quality in Health Care*. 2007. Volume 19, Number 6: pp. 349 – 357
